# Infectious complications following major heart surgery from the day of the surgery to hospital discharge

**DOI:** 10.1186/s12879-023-08972-9

**Published:** 2024-01-11

**Authors:** Maria Jesús Pérez-Granda, José María Barrio, Gregorio Cuerpo, Maricela Valerio, Patricia Muñoz, Javier Hortal, Angel González Pinto, Emilio Bouza, On behalf of the Cardiovascular Infection Study Group, Begoña Quintana, Alejandro Garrido Sánchez, Mónica Barranco, Eduardo Sánchez Perez, Francisco Moraga, Alba López, Patricia Bono, Ignacio Fernández López, Guillermo Rodríguez Bernal, Enma Novoa, Roberto Hugo Rodríguez Abella, Manolo Ruiz, Alvaro Pedraz, Diego Monzón Diaz, Ramón Fortuny, Javier Rodríguez Lega, Maria Yolanda Villa Gallardo, Laura Diaz Calvo, Sara Solís Gallego, Carmen Garcia Mere, Alvaro Alvarez Tomás

**Affiliations:** 1 Clinical Microbiology and Infectious Diseases Department, Hospital General Universitario Gregorio Marañón, Doctor Esquerdo, 46, 28007 Madrid, Spain; 2 CIBER de Enfermedades Respiratorias-CIBERES (CB06/06/0058), Madrid, Spain; 3 Department of Nursing, School of Nursing, Physiotherapy and Podiatry, Universidad Complutense de Madrid, Madrid, Spain; 4 Department of Anesthesiology, Hospital General Universitario Gregorio Marañón, Madrid, Spain; 5 Department of Cardiac Surgery, Hospital General Universitario Gregorio Marañón, Madrid, Spain; 6 Instituto de Investigación Sanitaria Gregorio Marañón (IiSGM), Madrid, Spain; 7 Department of Medicine Department, School of Medicine, Universidad Complutense de Madrid, Madrid, Spain

**Keywords:** Major heart surgery, Postoperative infections, Surgical site infections, Pneumonia, Ventilator associated pneumonia, Bloodstream infections, Mortality, Nosocomial infections

## Abstract

**Background:**

At some point in their lives, many people will require major heart surgery (MHS). Patients are generally older adults with various risk factors for infection. However, the incidence of infection after MHS is poorly known, as reported infection data are frequently biased due to different factors like the surgical procedure, postoperative timing, and infectious syndromes or etiologic agents, among others. In addition, most patient data are retrospectively obtained.

**Purpose and methods:**

Data were prospectively collected regarding the incidence of all nosocomial infections produced from the time of surgery to hospital discharge in a cohort of 800 adults consecutively undergoing a MHS procedure.

**Results:**

During postoperative hospitalization, 124 of the 800 participants developed one or more infections (15.5%): during their ICU stay in 68 patients (54.8%), during their stay on the general ward post ICU in 50 (40.3%), and during their stay in both wards in 6 (4.8%). The most common infections were pneumonia (related or not to mechanical ventilation), surgical site and bloodstream. As etiological agents, 193 pathogens were isolated: mostly Gram-negative bacilli (54.4%), followed by Gram-positive bacteria (30%), viruses (4.6%) and fungi (1.5%). In our cohort, all-cause mortality was recorded in 33 participants (4.1%) and 9 infection-related deaths (1.1%) were produced. Among subjects who developed infections, overall mortality was 13.7% and in those who did not, this was only 2.3%.

**Conclusion:**

Infection following MHS remains frequent and severe. Our data suggest that hospital-acquired infection studies should consider episodes of infection in all populations during their entire hospital stay and not only those related to specific clinical syndromes or acquired while the patient is in intensive care.

## Background

A high number of patients undergoes one or more major heart surgery (MHS) procedures annually (29, 319 procedures in 2021 in Spain). Patients are usually older persons with numerous risk factors for acquiring an infection. The overall infection risk has been calculated as 5.9 to 20% of all patients undergoing MHS [1, 2]. However, many of the data reported are biased for several reasons including the reporting of only one particular type of infection such as surgical site infection [3–6], ventilator-associated pneumonia [7–9] or catheter-related bloodstream infection [10]. Other reports focus on the causative agents such infections produced by *S. aureus* or *Mycobacterium chimera* [11–13]. In other studies, the selective factor is the surgical procedure itself including infections following revascularization surgery or valve surgery [14]. Moreover, many infection studies seem to consider only the early postoperative period, when the patient in is a post-surgical ICU. In effect, infections occurring in the postoperative period when the patient has left the ICU have been less studied and are not usually compared with those occurring in the ICU.

In 2006, we reported the results of a hospital-acquired infection study performed in several European MHS-ICUs via a questionnaire sent to 17 hospitals. Overall, this retrospective study revealed that 9.9% of operated patients developed one or more nosocomial infections during their ICU stay along with an associated median mortality of 4.7% [1].

The aim of the present study was to determine the incidence, type, etiology and prognosis of infections produced in a cohort of adults undergoing MHS based on data prospectively collected from the day of surgery to hospital discharge. Our main objective was to better estimate the actual risk of infection and the type of infections produced during the entire period of hospitalization after MHS.

## Material and methods

### Hospital setting and patients

Our institution is a tertiary care hospital with approximately 1300 beds and around 50,000 admissions/year. More than 500 MHS procedures are performed annually in adults at the Dept. of Cardiovascular Surgery, a large referral unit.

### Study design

This was a prospective observational cohort study of adult patients undergoing MHS at our center over the inclusive period October 10, 2019 to December 17, 2021.

### Primary endpoint

The primary endpoint was the incidence of postoperative infections acquired before hospital discharge after MHS.

### Secondary endpoints

As secondary endpoints, we established the incidences of different types of infection, causative microorganisms, risk factors for infection, and infection-related mortality.

### Follow-up

Physicians from the Depts. of Anesthesia and Infectious Diseases monitored patients daily to check for the presence of infection. Our infection control team is multidisciplinary including physicians and nurses from the ICU, microbiologists, infectious disease specialists, and health care workers from the Dept. of Preventive Medicine. In adult patients undergoing MHS, data are regularly and prospectively collected into a database. Patient follow up at the center was no less than 4 months after MHS.

### Microbiological samples

All microorganisms were identified using standard methods, and antimicrobial susceptibility was tested according to Clinical and Laboratory Standards Institute recommendations.

All patients were tested for the presence of SARS-CoV 2 prior to surgery even if they were asymptomatic, and also after surgery to exclude cases of nosocomial acquisition. All respiratory specimens from symptomatic patients were tested for SARS-CoV 2.

### Definitions

The definitions of infections were based on the criteria issued by the CDC, particularly the definitions of hospital-acquired pneumonia (HAP), ventilator-associated pneumonia (VAP), bacteremia, catheter-related bloodstream infection (CR-BSI), surgical site infection, mediastinitis and urinary tract infection (UTI) [15–18].

### Preventive measures

All patients scheduled for an MHS procedure were routinely examined to determine their nasal *Staphylococcus aureus* carrier status by PCR testing and/or culture on traditional media. Colonized patients were decontaminated with nasal mupirocin. Daily hygiene was performed with chlorhexidine impregnated wipes.

The bundle of measures for prevention of pneumonia included aspiration of subglottic secretions using a TaperGuard Evac endotracheal tube (Mallinckrodt, St Louis, Mo), with cuff pressure maintained at 20 to 30 mmHg, continuous monitoring with the patient in a semirecumbent position (30°–45°), and oral hygiene with chlorhexidine. Patients who remained under mechanical ventilation (MV) for more than 48 h were systematically subjected to selective digestive decontamination (SDD) without the contribution of systemic antibiotics. Was systematically applied to patients who remained under mechanical ventilation (MV) for more than 48 hours [19].

Catheter care included the following: daily recording of the need for catheter use, daily monitoring of the insertion site, skin disinfection with 2% alcoholic chlorhexidine, connector disinfection with 70% alcohol wipes before use, replacement of gauze/transparent/chlorhexidine dressing according to international guidelines, and use of split-septum closed connectors (CLAVE, ICU Medical, Inc., San Clemente, CA, USA) and replacement of continuous infusion systems every 7 days (except for parenteral nutrition every 24 h and propofol every 12 h).

Further information recorded daily was the need for a catheter. Patients were catheterized using lose drainage systems with the drainage bag always placed below the level of the bladder. Hygiene and care measures on the urethral meatus were performed daily. Patients started wearing a sternum support vest 24 h after surgery when possible [20, 21]. Antibiotic prophylaxis for surgery consisted of 2 g of cefazolin administered before surgery and every 8 h thereafter for a total of three doses (if allergic to cefazolin 1 g of vancomycin was given before surgery and two further doses every 12 h thereafter).

### Statistical analysis

Qualitative variables are provided with their frequency distributions. Quantitative variables are given as the mean and standard deviation (SD), and median and inter-quartile range (IQR) if their distribution was skewed. Continuous variables were compared using the *t* test for normally distributed variables, or median test for non-normally distributed variables. Categorical variables were compared using the chi-square or Fisher’s exact test.

Variables included in the multivariate logistic analysis were those emerging as significant in the analysis and/or those considered clinically relevant [22].

Following the multivariate analysis, a risk score model was constructed to identify participants at a higher risk for intra-hospital infection. This model was subjected to ROC curve analysis and a cutoff value searched for to determine the accuracy and optimal threshold of high-risk patients for infection. Next, we calculated sensitivity, specificity, positive and negative predictive values, and likelihood ratios for different scores. The risk of infection according to the onset time of the first episode of nosocomial infection was assessed through Kaplan-Meier analysis.

All statistical tests were performed using the software packages SPSS 21.0 and Epidat 2.1. Significance was set at *p* < 0.05.

## Results

### Study population

Participants were 800 subjects consecutively undergoing MHS who gave their written informed consent to participate. After enrolment, participants were prospectively followed until hospital discharge. Median participant age was 64 years (IQR 56–73.7); 68.3% were men (Table 1).

**Table 1:** Patient characteristics

Patients	Total *N* = 800	WITH INFECTION *N* = 124	WITHOUT INFECTION *N* = 676	*p*
Median age in years (IQR)	64.0 (56.00–73.75)	64.0 (58.0–73.0)	64.0 (56.0–74.0)	0.893
**Sex (%)**				0.970
Male	546 (68.3)	87 (70.1)	459 (67.8)	
Female	254 (31.8)	37 (29.8)	217 (32.1)	
Obesity (body mass index > 30) (%)	181 (22.62)	35 (28.22)	146 (21.59)	0.128
**Underlying conditions (%)**				
Myocardial infarction	108 (13.5)	20 (16.1)	89 (13.1)	0.393
Congestive heart failure	181 (22.6)	53 (42.7)	128 (18.9)	< 0.001
Central nervous system disease	94 (11.8)	19 (15.3)	75 (11.0)	0.175
Chronic obstructive pulmonary disease	105 (13.1)	24 (19.3)	81 (11.9)	0.030
Renal dysfunction	54 (6.8)	20 (16.1)	34 (5.0)	< 0.001
Diabetes mellitus	199 (24.9)	45 (36.2)	154 (22.7)	0.002
Peptic ulcer disease	60 (7.5)	11 (8.8)	49 (7.2)	0.577
Peripheral vascular disease	59 (7.4)	13 (10.4)	46 (6.8)	0.188
Tumor	73 (9.1)	12 (9.6)	61 (9.0)	0.865
**Positive asal Samples,** ***n*** **= 195**				1.000
Nasal-MSSA	187 (23.3)	29 (23.3)	158 (23.3)	
Nasal MRSA	8 (1.0)	1 (0.8)	7 (1.0)	
**Urgent surgery (%)**	18 (2.25)	7 (5.64)	11 (1.62)	0.013
Apache, mean (SD)	7.49 (3.04)	8.7 (2.9)	7.2 (3.0)	< 0.001
Charlson, mean (SD)	1.56 (1.70)	2.4 (1.9)	1.4 (1.6)	< 0.001
EuroScore, median (IQR)	6.0 (3.09–8.00)	7.0 (5.0–9.0)	6.0 (3.0–8.0)	< 0.001
Risk, EuroScore, median (IQR)	5.04 (2.54–10.71)	7.5 (3.5–15.9)	4.7 (2.4–9.3)	0.001
EuroScore II, median (IQR)	1.58 (0.90–3.12)	2.7 (1.2–7.4)	1.5 (0.8–2.8)	< 0.001
**Type of surgery (%)**				
Valve replacement	393 (49.1)	63 (50.8)	330 (48.8)	0.697
CABG	246 (30.7)	37 (29.8)	209 (30.9)	0.916
Mixed (valve and CABG)	49 (6.1)	10 (8.0)	39 (5.7)	0.409
Aortic no valve + Aortic and valve	39 (4.8)	4 (3.2)	35 (5.1)	0.496
Congenital	27 (3.3)	1 (0.8)	26 (3.8)	0.104
Other	46 (5.7)	8 (6.4)	38 (5.6)	0.677
**Surgical data**				
Median (IQR) CPBT (min)	127.00 (90.00–167.00)	135.0 (91.0–186.0)	125.0 (90.0–166.0)	0.055
Median (IQR) aortic cross-clamp time (min)	84.00 (58.00–112.00)	89.0 (56.0–124.0)	83.0 (58.7–110.0)	0.568
**Re-intervention (%)**	52 (6.5)	17 (13.7)	35 (5.1)	0.001
**Transfusion (%)**	444 (55.5)	91 (73.4)	353 (52.2)	< 0.001
**Packing (%)**	30 (3.8)	17 (13.7)	13 (1.9)	< 0.001
Median length of hospital stay in days (IQR)	15.0 (10.0–24.0)	32.0 (23.2–51.2)	14.0 (10.0–20.0)	< 0.001
Median length of ICU stay in days (IQR)	4.0 (3.0–6.0)	7.5 (5.0–16.0)	4.0 (2.0–6.0)	< 0.001
Median length of preoperative stay in days (IQR)	2.0 (1.0–7.0)	4.0 (1.0–13.0)	1.5 (1.0–6.0)	0.001
Days of MV, mean (IQR)	2.6 (10.4)	9.7 (25.2)	1.3 (1.3)	< 0.001
Patients with MV > 48 h (%)	101 (12.6)	51 (41.1)	50 (7.4)	< 0.001
Reintubation (%)	38 (4.7)	24 (19.3)	14 (2.0)	< 0.001
DDDs, mean (SD)	16.3 (31.0)	21.9 (37.8)	7.9 (14.1)	0.001
Mortality (%)	33 (4.1)	17 (13.7)	16 (2.3)	< 0.001

*IQR* interquartile range, *SD* standard deviation, *CABG* coronary artery by-pass grafting, *CPBT* cardiopulmonary by-pass time, *MV* mechanical ventilation

Patient comorbidities (Table 1) were classified using the modified Charlson method, yielding a mean index of 1.56 (SD 1.70). Diabetes mellitus was the most frequent underlying disease affecting 24.9% of the cohort, followed by congestive heart failure (22.6%), previous ischemic heart disease (13.5%) and COPD (13.1%). Surgical risk scores for the cohort were (medians): EuroScore = 6 (IQR 3.09–8), RISK Euroscore = 5.04 (IQR 2.54–10.71) and Euroscore 2 = 1.58 (IQR 0.90–3.12).

### Types of surgery

Of all the MHS interventions conducted, 49.1% were heart valve surgery, 30.7% were coronary artery bypass grafting (CABG) procedures and the remaining 6.1% were other types of surgeries or mixed surgeries (valve and heart). Median extracorporeal circulation time was 127 min (range 90–167).

In this cohort, 6.5% (52/800) required one or more re-interventions and 55.4% (444/800) of those operated on required a blood transfusion during the first 24 h.

### Hospital stay

The median length of hospital stay (Table 1) was 15 days for overall stay (IQR 10–25) and 4 days (IQR 3–6) for postoperative ICU stay (IQR 8–18). In our cohort, 12.6% required more than 48 h of mechanical ventilation.

### Infections postsurgery and timing

Of the 800 patients who underwent MHS, 124 (15.5%) had one or more hospital-acquired infections during their hospital stay: 68 (54.8%) had one or more infections during their stay in the ICU (54.8%), 50 during their stay on the ward post ICU (40.3%), and 6 (4.8%) had infections in both wards.

### Types of infection

Table 2 shows the episodes of infection recorded and their etiology.

**Table 2:** Microorganisms causing nosocomial infection

Microorganisms	Bacteremia 12 patients (13 episodes)	CR-BSI 4 patients	VAP 27 patients 29 episodes (1 polimicrobian)	Tracheobronchitis 18 patients 21 episodes (2 polimicrobian)	Pneumonia not associated with MV 25 patients 26 episodes (2 polimicrobians)	Surgical Wound 37 patients 39 episodes		Urinary Tract Infection 30 patients 31 episodes (2 polimicrobians)	Invasive aspergillosis	CDI (11 patients)	Covid-19	Total
						Superficial Surgical Wound 24 patients 25 episodes (4 policrobians)	Mediastinitis 13 patients 14 episodes (2 policrobians)					
**Gram positive**												**47**
*S.epidermidis/* CoNS		3				15	9					27
*Staphylococcus aureus*			2	1		3	2					8
*Enterococcus* species	2			1		1		2				6
*Corynebacterium spp*						1	1					2
*Others*	1					1	2					4
**Gram negative**												**105**
*Serratia marcescens*	3		7	8	2	2		3				25
*Klebsiella* spp.				2	3			7				12
*Pseudomonas aeruginosa*	1		4	1	1	3		6				16
*Proteus mirabilis*	2		2		1		1	9				15
*Enterobacter species*	1		2	1	2			3				9
*Escherichia coli*	3		5		2	2	1	2				15
*H.influenzae*			1	2	4							7
*Others*			3	3								6
**Fungi**												**3**
*Candida spp*		1										1
*Aspergillus*									2			2
**Virus**												**9**
*Covid-19*					3						4	7
*Influenza A*					2							2
**C.*difficile***										11		**11**
**No microorganisms**			5	4	8	1						**18**
**TOTAL**												**193**

*CDI Clostridioides difficile* infection

There were 29 episodes of mechanical ventilation-associated pneumonia (affecting 27 patients) leading to an incidence density of 13.5 episodes/1000 days of ventilation. There were also 21 episodes of tracheobronchitis during mechanical ventilation (26.2/1000 admissions). In addition, 26 episodes of pneumonia not related to mechanical ventilation (32.5/1000 admissions) were diagnosed, and these episodes occurred mostly outside the ICU. Superficial wound infection was observed in 24 patients (25 episodes). There were 14 episodes (13 patients) classified as mediastinitis (1.75%). We recorded 13 episodes of bloodstream infection, 4 related to endovascular catheters (0.5/1000 admissions), none of which were classified as postsurgical endocarditis. There were also 31 (30 patients) episodes of urinary tract infection (38.75/1000 admissions) recorded.

Eleven episodes of hospital-acquired infection (1.4%) were attributed to *Clostridioides difficile* infection (CDI).

Further infections recorded were: 1 episode of cholangitis, 2 of invasive pulmonary aspergillosis (2 patients) and 7 patients had a hospital-acquired mild COVID infection (4 with pneumonia and 3 without pneumonia).

### Causative microorganisms

Of all infections occurring in the postoperative period after heart surgery (191 episodes, 193 microorganisms identified), 18 could not be etiologically identified. The remaining episodes (Table 2) were mainly caused by Gram-negative bacilli (105–54.4%) and Gram-positive bacteria (47–24.3%). Cytomegalovirus (CMV) reactivation occurred in 25 patients (3.1%), but as the virus was not hospital-acquired, these cases are not included in the table.

### *S. aureus* nasal carrier status

Of the whole cohort, 195 patients were identified as nasal carriers of *S. aureus* and the remaining 605 (75.6%) returned a negative nasal PCR result for *S. aureus* on admission. *Staphylococcus aureus* infection rates in patients who were nasal carriers and non-carriers of this bacterium were respectively 3/195 (1.5%) and 5/605 (0.8%; *p* = 0.411).

### Risk factors for infection

Table 1 compares the characteristics of participants with and without postoperative nosocomial infections. Those with one or more hospital-acquired infections had more frequent heart failure, renal dysfunction, a higher APACHE score and a higher Charlson’s comorbidity index. These patients received more transfusions and required sternal packing more frequently. In our univariate analysis, it emerged that infected patients spent more days on mechanical ventilation, required reintubation on more occasions, consumed more defined daily doses of antibiotics, and their ICU and hospital stays were longer.

Multivariate analysis identified several independent variables associated with hospital-acquired infection: congestive heart failure, renal dysfunction, preoperative hospital stay > 3 days, mechanical ventilation > 48 h and reintubation (Table 3).

**Table 3:** Multivariate analysis of risk factors for nosocomial infection in patients who underwent MHS

Variables	*p*	OR (95%CI)
**Chronic obstructive pulmonary disease**	0.133	1.56 (0.87–2.80)
**Congestive heart failure**	**0.008**	1.88 (1.18–3.02)
**Renal dysfunction**	**0.028**	2.13 (1.08–4.20)
**Diabetes**	0.581	1.14 (0.70–1.86)
**Preoperative length of stay > 3 days**	**0.063**	1.51 (0.97–2.35)
**Transfusion**	0.205	1.37 (0.84–2.24)
**Emergency surgery**	0.704	1.26 (0.38–4.18)
**Inotropic support after surgery**	0.414	1.55 (0.54–4.50)
**Mechanical ventilation > 48 hours**	**< 0.001**	4.19 (2.40–7.33)
**Re-intubation**	**0.009**	3.08 (1.32–7.15)
**Re-intervention**	0.943	1.03 (0.48–2.20)

**Fig. 1:**
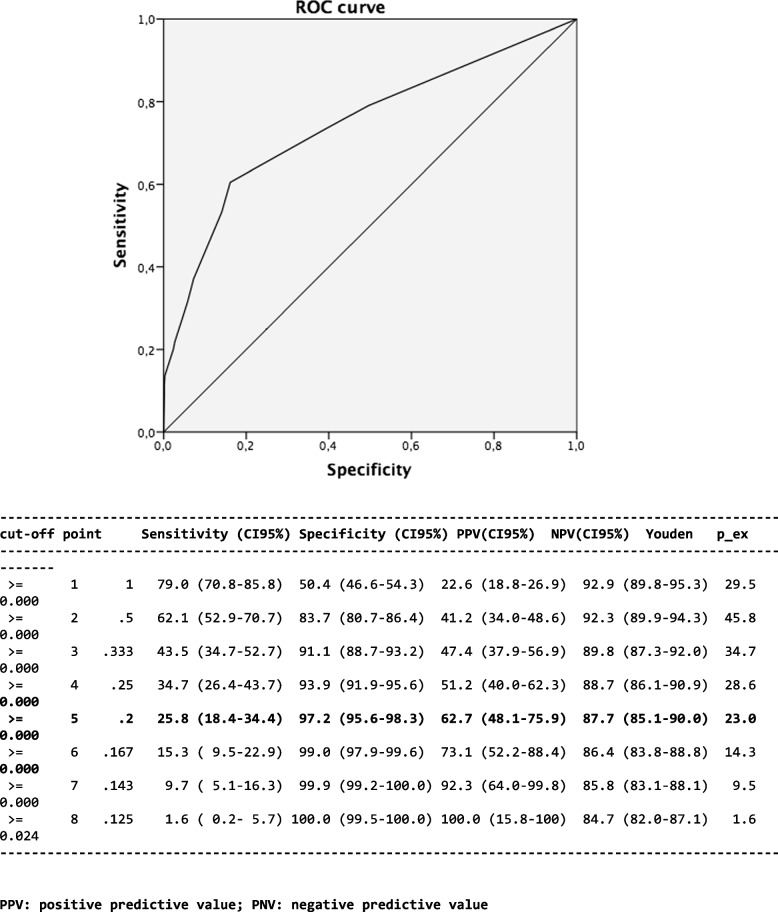
ROC curve analysis and a cutoff value searched for to determine the accuracy and optimal threshold of high-risk patients for infection

Table 4 shows the logistic regression model of coefficients converted into a simplified score. One of our ROC curves (Fig. 1) yielded a specific value of 7 as the cutoff with an AUC = 0.745 (CI 95%).

**Table 4:** Logistic regression model coefficients converted to calculate a simplified score

Variables	*p*	OR (95%CI)	Score
**Congestive heart failure**	**< 0.001**	2.09 (1.33–3.30)	**1**
**Renal dysfunction**	**0.001**	2.27 (1.15–4.45)	**1**
**Preoperative length of stay > 3 days**	**0.040**	1.56 (1.02–2.40)	**1**
**Mechanical ventilation > 48 hours**	**< 0.001**	4.84 (2.84–8.25)	**3**
**Re-intubation**	**0.005**	3.29 (1.44–7.51)	**2**

**Fig. 2:**
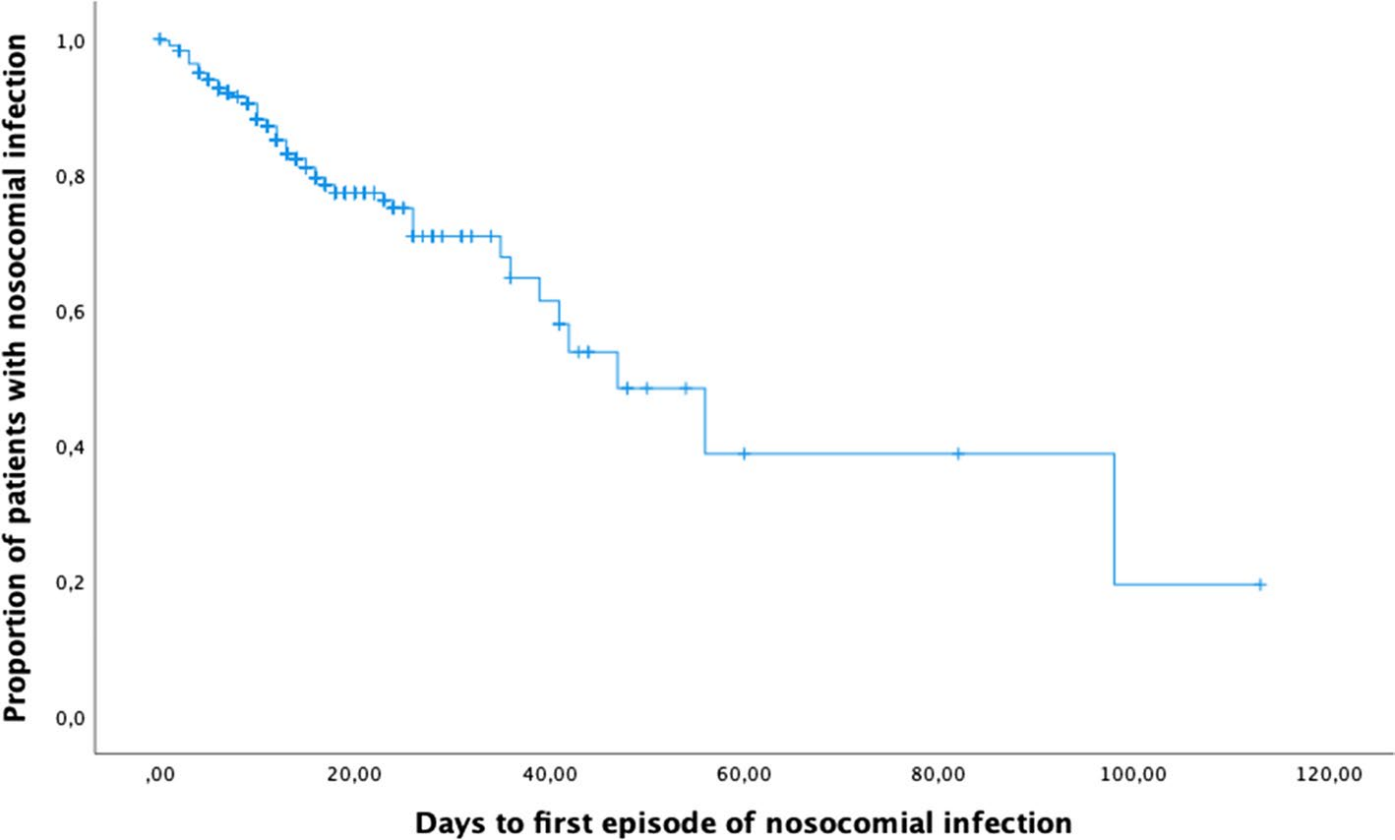
Kaplan-Meier analysis of the risk of infection according to the onset time of the first episode of nosocomial infection

Our Kaplan-Meier assessment of infection risk according to timing of the first episode of hospital-acquired infection is depicted in Fig. 2.

### Prognosis

Overall mortality in all 800 surgical patients was 33 (4.1%). Of these, 27 (81.8%) patients died during ICU stay and the remaining 6 (18.2%) patients during their postoperative stay in general wards after ICU discharge. Of these 33 deaths, 9 (27.2%) were primarily due to infection. Overall mortality was higher (13.7%) in patients who developed infections than in those who did not (2.3%).

## Discussion

Our work shows that when all infections recorded after heart surgery up until hospital discharge are considered, figures are higher at 15.5% than those commonly reported in the literature. It also reveals that MHS continues to give rise to a high incidence of infection and a not insignificant mortality rate. This study also identifies a need for global figures that offer a real perspective of the risk of infection.

Following MHS, infection risk estimates are often biased as different reports usually consider only certain factors such as a given period of postoperative stay, type of surgery, type of infection or even certain groups of causative microorganisms. For instance, the reported incidence of surgical wound infection ranges from 2.6 to 5.5% and is higher in heart transplant recipients [5, 23–25].

In another study, the incidence of pneumonia after heart surgery was estimated at 3.5% of 7439 consecutive patients, including 120 (47%) cases of non-VAP episodes [26]. Eleven studies on ventilator associated pneumonia (VAP) after MHS included in a meta-analysis reported a figure of 21 episodes/1000 ventilator-days. Prevalence was 6.4% of all patients and 35.2% of patients who were on mechanical ventilation for more than 48 h [27]. According to more recent data, the incidence density was of 17.7 episodes per 1000 ventilator-days [28]. Our findings show that if infections are prospectively recorded without any bias, the incidence of infection after this “clean surgery” procedure remains high.

Numerically, infections occur almost as frequently in the postoperative ICU period as in the time spent in general wards, although it is true that the severity and risk of death of the infections acquired are higher in the former [29, 30].

In our analysis of causative microorganisms and another study [31], Gram-negative bacteria emerged as predominant, representing 54% of all causes of infection. However, in a study focusing on local infectious complications of heart surgery the predominance of Gram-positive cocci was observed [32]. Fungal infections following MHS are much less common.

Our data indicate the continued need for *S. aureus* colonization screening in patients scheduled to undergo MHS.

It is well known that VAP, SSI and catheter-related bloodstream infections are major concerns following MHS [30, 33]. However, a more detailed look at our findings reveals that almost half of all post–MHS episodes of pneumonia cannot be ascribed to mechanical ventilation but rather they occur after extubation and transfer of the patients to a general ward. The numerous episodes of urinary tract infection detected in patients requiring bladder catheterization after surgery is also striking. The need for catheterization should be re-examined by means of a daily check list to reduce this risk factor.

We feel that comprehensive programs are needed in which the same team controlling infection in intensive care continues to follow these patients until they are discharged from hospital. These programs should target zero tolerance to these infections with education and other types of interventions.

The risk factors for infection in our patient subset were relatively well defined as in other studies and include surgery duration, heart failure, need for continuous veno-venous hemofiltration, longer mechanical ventilation, reintubation and tracheostomy, and delayed closure of the sternal wound [2, 4, 30, 31]. The model constructed and a cutoff value could be used in future investigations to implement preventive measures.

As we have previously observed, a very clear marker of these infections was the need for whatever reason to remain in the ICU for more than 72 hours after surgery. It is clear that this subgroup of patients requires special attention and interventions to prevent and lower the incidence of these infections [1, 22, 34, 35]. We are not aware, however, of programs that have specifically managed to reduce mortality due to infections attributed to a lengthened ICU stay.

Our mortality differences between infected and uninfected patients (23% vs. 2%) resemble those reported in another Spanish study in which rates were 18% versus 5% respectively [30].

The economic burden of hospital-acquired infection is another factor to consider if only to raise awareness in hospital managers so that control measures are justified. In Japan, the cost of VAP alone has been estimated at £15,124 compared to £6295 for non-ventilator-associated pneumonia. Infected patients were reported by the authors of this last study to cost 5 times more, and hospital stay was lengthened 9.3 ± 2.6 days [36].

The main limitation of our work was that it was a single-center study so its results may not be necessarily transferable to other institutions. Nevertheless, our main message remains that postoperative infection assessment after MHS should not discriminate between types of patients, types of infections, or times at which infection occurs. We should also mention that while our study period coincided with that of the COVID-19 pandemic, we found no difference in infection rates between the two periods.

## Conclusion

Most studies designed to analyze infections occurring after MHS fail to consider certain types of infection, types of patients, or times at which infection occurs. Accordingly, our study reveals a higher incidence of infections after MHS than generally reported in the literature. This information confirms that infections are still frequent and severe, and identifies an urgent need for standardized protocols so that infection rates can be effectively compared across different centers.

## Data Availability

All data generated or analyzed during this study are included in this article.

## Abbreviations

ICU: Intensive care unit
MHS: Major heart surgery
MV: Mechanical ventilation
VAP: Ventilator-associated pneumonia
SDD: Selective digestive decontamination
SD: Standard deviation
HAP: Hospital-acquired pneumonia
VAP: Ventilator-associated pneumonia
CR-BSI: Bacteremia, catheter-related bloodstream infection
SSI: Surgical site infection
MD: Mediastinitis
UTI: Urinary tract infection
ROC: Receiver Operating Characteristic
AUC: Area under the curve

## References

[1] Bouza E, Hortal J, Muñoz P, Pérez MJ, Riesgo MJ, Hiesmayr M (2006). Infections following major heart surgery in European intensive care units: there is room for improvement (ESGNI 007 study). J Hosp Infect..

[2] Michalopoulos A, Geroulanos S, Rosmarakis ES, Falagas ME (2006). Frequency, characteristics, and predictors of microbiologically documented nosocomial infections after cardiac surgery. Eur J Cardio-Thorac Surg : Off J Eur Assoc Cardio-Thorac Surg..

[3] Vitartaitė M, Vaičiulytė D, Venclovienė J, Širvinskas E, Bukauskienė R, Jakuška P (2021). Risk factors associated with an increased risk of deep sternal wound infections in patients after coronary artery bypass grafting and heart defect surgery. Heart Surg Forum..

[4] Jayakumar S, Khoynezhad A, Jahangiri M (2020). Surgical site infections in cardiac surgery. Crit Care Clin.

[5] Figuerola-Tejerina A, Rodríguez-Caravaca G, Bustamante-Munguira J, María San Román-Montero J, Durán-Poveda M (2016). Epidemiological surveillance of surgical site infection and its risk factors in cardiac surgery: a prospective cohort study. Rev Esp Cardiol (English ed)..

[6] Morikane K (2018). Epidemiology and risk factors associated with surgical site infection following surgery on thoracic aorta. Epidemiol Infect.

[7] Pérez-Granda MJ, Barrio JM, Muñoz P, Hortal J, Rincón C, Bouza E (2014). Impact of four sequential measures on the prevention of ventilator-associated pneumonia in cardiac surgery patients. Crit Care (London, England)..

[8] Fitch ZW, Whitman GJ (2014). Incidence, risk, and prevention of ventilator-associated pneumonia in adult cardiac surgical patients: a systematic review. J Card Surg.

[9] Le Guillou V, Tavolacci MP, Baste JM, Hubscher C, Bedoit E, Bessou JP (2011). Surgical site infection after central venous catheter-related infection in cardiac surgery. Analysis of a cohort of 7557 patients. J Hosp Infect.

[10] Pérez-Granda MJ, Barrio JM, Muñoz P, Hortal J, Rincón C, Rabadán PM (2014). Ethanol lock therapy (E-lock) in the prevention of catheter-related bloodstream infections (CR-BSI) after major heart surgery (MHS): a randomized clinical trial. PLoS One.

[11] Acosta F, Pérez-Lago L, Ruiz Serrano MJ, Marín M, Kohl TA, Lozano N (2018). Fast update of undetected Mycobacterium chimaera infections to reveal unsuspected cases. J Hosp Infect.

[12] Pongbangli N, Oniem N, Chaiwarith R, Nantsupawat T, Phrommintikul A, Wongcharoen W (2021). Prevalence of Staphylococcus aureus nasal carriage and surgical site infection rate among patients undergoing elective cardiac surgery. Int J Infect Dis : IJID : Off Publ Int Soc Infect Dis.

[13] Bouza E, Burillo A, de Egea V, Hortal J, Barrio JM, Vicente T (2020). Colonization of the nasal airways by Staphylococcus aureus on admission to a major heart surgery operating room: a real-world experience. Enferm Infecc Microbiol Clin (English ed)..

[14] Abuzaid AA, Zaki M, Al TH (2015). Potential risk factors for surgical site infection after isolated coronary artery bypass grafting in a Bahrain cardiac Centre: a retrospective, case-controlled study. Heart Views: Off J Gulf Heart Assoc..

[15] Garner JS, Jarvis WR, Emori TG, Horan TC, Hughes JM (1988). CDC definitions for nosocomial infections, 1988. Am J Infect Control.

[16] Horan TC, Andrus M, Dudeck MA (2008). CDC/NHSN surveillance definition of health care-associated infection and criteria for specific types of infections in the acute care setting. Am J Infect Control.

[17] Horan TC, Gaynes RP, Martone WJ, Jarvis WR, Emori TG (1992). CDC definitions of nosocomial surgical site infections, 1992: a modification of CDC definitions of surgical wound infections. Am J Infect Control.

[18] Borchardt RA, Tzizik D (2018). Update on surgical site infections: the new CDC guidelines. JAAPA : Off J Am Acad Physician Assist.

[19] Pérez-Granda MJ, Barrio JM, Hortal J, Burillo A, Muñoz P, Bouza E (2018). Impact of selective digestive decontamination without systemic antibiotics in a major heart surgery intensive care unit. J Thorac Cardiovasc Surg.

[20] Vos RJ, Van Putte BP, Kloppenburg GTL (2018). Prevention of deep sternal wound infection in cardiac surgery: a literature review. J Hosp Infect.

[21] Tewarie LS, Menon AK, Hatam N, Amerini A, Moza AK, Autschbach R (2012). Prevention of sternal dehiscence with the sternum external fixation (Stern-E-Fix) corset--randomized trial in 750 patients. J Cardiothorac Surg.

[22] Hortal J, Giannella M, Pérez MJ, Barrio JM, Desco M, Bouza E (2009). Incidence and risk factors for ventilator-associated pneumonia after major heart surgery. Intensive Care Med.

[23] Sommerstein R, Marschall J, Kuster SP, Troillet N, Carrel T, Eckstein FS (2019). Cardiovascular daytime varying effect in cardiac surgery on surgical site infections and 1-year mortality: a prospective cohort study with 22,305 patients. Infect Control Hosp Epidemiol.

[24] Morikane K, Honda H, Yamagishi T, Suzuki S (2015). Differences in risk factors associated with surgical site infections following two types of cardiac surgery in Japanese patients. J Hosp Infect.

[25] Rodrigues JA, Ferretti-Rebustini RE, Poveda VB (2016). Surgical site infection in patients submitted to heart transplantation. Rev Lat Am Enfermagem.

[26] Allou N, Allyn J, Snauwaert A, Welsch C, Lucet JC, Kortbaoui R (2015). Postoperative pneumonia following cardiac surgery in non-ventilated patients versus mechanically ventilated patients: is there any difference?. Crit Care (London, England)..

[27] He S, Chen B, Li W, Yan J, Chen L, Wang X (2014). Ventilator-associated pneumonia after cardiac surgery: a meta-analysis and systematic review. J Thorac Cardiovasc Surg.

[28] Hassoun-Kheir N, Hussein K, Abboud Z, Raderman Y, Abu-Hanna L, Darawshe A, et al. Risk factors for ventilator-associated pneumonia following cardiac surgery: case-control study. J Hosp Infect. 2020;105 PubMed PMID: 32283174. Epub 2020/04/14. Eng. 10.1016/j.jhin.2020.04.009 32283174

[29] Mazzeffi M, Gammie J, Taylor B, Cardillo S, Haldane-Lutterodt N, Amoroso A (2017). Healthcare-associated infections in cardiac surgery patients with prolonged intensive care unit stay. Ann Thorac Surg.

[30] de la Varga-Martínez O, Gómez-Sánchez E, Muñoz MF, Lorenzo M, Gómez-Pesquera E, Poves-Álvarez R (2021). Impact of nosocomial infections on patient mortality following cardiac surgery. J Clin Anesth.

[31] Liu Z, Zhang X, Zhai Q. Clinical investigation of nosocomial infections in adult patients after cardiac surgery. Medicine. 100(4):e24162. PubMed PMID: 33530207. Pubmed Central PMCID: PMC7850681. Epub 2021/02/04. Eng. 10.1097/MD.0000000000024162 PMC7850681 33530207

[32] Stepin AV (2022). Local infectious complications in cardiac surgery: etiology and the role of antimicrobial prophylaxis. Khirurgiia..

[33] Chen LF, Arduino JM, Sheng S, Muhlbaier LH, Kanafani ZA, Harris AD (2012). Epidemiology and outcome of major postoperative infections following cardiac surgery: risk factors and impact of pathogen type. Am J Infect Control.

[34] Bouza E, Hortal J, Muñoz P, Pascau J, Pérez MJ, Hiesmayr M (2006). Postoperative infections after major heart surgery and prevention of ventilator-associated pneumonia: a one-day European prevalence study (ESGNI-008). J Hosp Infect.

[35] Bouza E, de Alarcón A, Fariñas MC, Gálvez J, Goenaga M, Gutiérrez-Díez F, et al. Prevention, diagnosis and Management of Post-Surgical Mediastinitis in adults consensus guidelines of the Spanish Society of Cardiovascular Infections (SEICAV), the Spanish Society of Thoracic and Cardiovascular Surgery (SECTCV) and the biomedical research Centre network for respiratory diseases (CIBERES). J Clin Med. 2021;10(23) PubMed PMID: 34884268. Pubmed Central PMCID: PMC8658224. Epub 2021/12/11. Eng. 10.3390/jcm10235566 PMC8658224 34884268

[36] Kobayashi J, Kusachi S, Sawa Y, Motomura N, Imoto Y, Makuuchi H (2015). Socioeconomic effects of surgical site infection after cardiac surgery in Japan. Surg Today.

